# Catatonic features in adolescents with schizophrenia with and without a comorbid pervasive developmental disorder

**DOI:** 10.1186/1753-2000-8-16

**Published:** 2014-05-23

**Authors:** Petra Waris, Nina Lindberg, Kirsi Kettunen, Jari Lipsanen, Pekka Tani

**Affiliations:** 1 Institute of Behavioural Sciences, University of Helsinki, PO Box 9, 00014 Helsinki, Finland; 2 Department of Psychiatry, Helsinki University, PO Box 3, 00014 Helsinki, Finland; 3 Department of Forensic Psychiatry, Kellokoski Hospital, 04500 Kellokoski, Finland; 4 Department of Adolescent Psychiatry, Kellokoski Hospital, 04500 Kellokoski, Finland; 5 Department of Psychiatry, Helsinki University Central Hospital, PO Box 442, 00029 HUS Helsinki, Finland

**Keywords:** Catatonia, Schizophrenia, Pervasive developmental disorder, Adolescence

## Abstract

**Background:**

Catatonia has been associated with both schizophrenia and pervasive developmental disorders. The aim of this study was to evaluate catatonic features among adolescents suffering from schizophrenia. Further, we compared these features between adolescents with a comorbid pervasive developmental disorder and those without one. Finally, we wanted to compare the profile of catatonia-like features of our schizophrenia patients to that described earlier among persons with autism spectrum disorders.

**Methods:**

The study comprised a consecutive sample of 18 adolescents with schizophrenia (mean age 15.6 years, SD 1.4) and their families. Diagnosis of schizophrenia was assessed with the Schedule for Affective Disorders and Schizophrenia for School-Aged Children – Present and Life-Time (K-SADS-PL) for the DSM-IV. The Diagnostic Interview for Social and Communication Disorders version 11 was used to assess catatonic features.

**Results:**

All adolescents with schizophrenia had showed some lifetime catatonic features. Approximately 78% of them had already expressed these features before the age of 10. The number of catatonic features before the age of 10 was significantly higher among the adolescents with a comorbid pervasive developmental disorder compared to those without one. The numbers of catatonic features after the age of 10 did not significantly differ between the two groups. Over three-quarters of schizophrenia patients shared four lifetime catatonic features: “lacks facial expression”, “odd intonation”, “poor eye contact” and “lack of cooperation”.

**Conclusions:**

Adolescent schizophrenia patients with a comorbid pervasive developmental disorder show many catatonic features in childhood whereas those without one seem to develop these features first in adolescence. Catatonic features exhibited by adolescents with schizophrenia resemble those described among persons with pervasive developmental disorders without schizophrenia.

## Background

Schizophrenia is a severe, pervasive mental disorder that is characterized by positive symptoms such as hallucinations, delusions and disorganized speech in addition to negative symptoms including marked apathy, paucity of speech, and blunting or incongruity of emotional responses. The onset of schizophrenia before the age of 18 is commonly categorized as early-onset schizophrenia. This form of schizophrenia can have either an acute or gradual onset. The onset of schizophrenia before the age of 13 is considered as childhood-onset schizophrenia, which in most cases shows an insidious onset [1]. The prevalence of childhood-onset schizophrenia is less than one in 10 000 children, but the prevalence of schizophrenia-related disorders among adolescents is about 1–2% [2,3].

Catatonia refers to a motor and mood dysregulation syndrome that includes motor signs such as stupor, mutism, negativism, grimacing, stereotypies and echopraxia/echolalia [4-8]. Originally, catatonia was classified as a subtype of schizophrenia. During the last decades, it has been associated with a wide range of medical disorders, both somatic and psychiatric in character [9]. In the DSM-5 classification, catatonia can be associated with another mental disorder, emerge in connection to another medical condition, or remain unspecific [9]. Besides the clinical diagnosis of catatonia, a wide range of chronic, less severe, and sometimes very subtle symptoms of posture, movement, speech, and behavior are considered to be catatonic in nature [10,11]. The term “catatonia spectrum” is used to cover the whole range of these manifestations [10].

Studies on adult patients with schizophrenia-related and mood disorders have reported incidences of catatonia that varied between10–38% [4,7,8]. However, despite that catatonia also occurs among children and adolescents [12], it has been sparsely studied among this age group. In a study by Thakur et al. [13], 17.7% of the children and adolescents who suffered from affective and non-affective psychotic disorders showed at least two signs of catatonia. Green et al. [14] reported that 31.6% of 38 hospitalized children with schizophrenia manifested catatonic features.

Pervasive developmental disorders (PDDs), affect 0.6–1% of the general population [15], and share a core triad of abnormalities: 1. qualitative impairments in reciprocal social interactions, 2. qualitative impairments in verbal and non-verbal communication, and 3. restricted social imagination with repetitive and stereotyped patterns of interests and behaviour [16]. Catatonic features have also been related to PDDs [17]. Wing and Shah [11] studied catatonia-like features in 200 children and adults with autism spectrum disorders (a broad equivalent of PDDs). They found that most of the participants had displayed some catatonic features during their lifetime. The items which occurred in more than three-quarters of the participants were: lack of facial expression, delayed echolalia, odd intonation, poor eye contact, and lack of cooperation. A prospective population-based follow-up study of 120 individuals with autism conducted by Billstedt et al. [18] reported that approximately 12% of the autistic persons met the criteria for catatonia during their adolescence.

Recently, Shorter and Wachtel [19] described historical patient vignettes in which catatonia, autism and psychosis were all present at the same time. According to the authors, these three diagnoses may represent three different manifestations of the same underlying brain disorder. Unfortunately, this “iron triangle” has been largely overlooked because its clinical features are conventionally seen as separate disorders. Yet, recognition of a mixed form of catatonia, autism and psychosis has important implications for both diagnosis and treatment.

The principal aim of the present study was to evaluate catatonic features among a consecutive sample of adolescents who were suffering from schizophrenia. Since both neuroimaging and genetic studies have reported that there is an overlap of symptoms between schizophrenia and PDDs [20,21], our second aim was to compare the nature and number of catatonic features between schizophrenia patients with a comorbid PDD and those without one. Further, we wanted to compare the profile of catatonia-like features of our schizophrenia patients to that described earlier among persons with autistic spectrum disorders without schizophrenia [11]. Our hypotheses were that adolescents suffering from schizophrenia manifest catatonic features, and that these features are more frequent among patients with a comorbid PDD. We also hypothesized that the profile of catatonia-like features of adolescents suffering from schizophrenia resembles that reported among autistic persons.

## Materials and methods

### Participants and procedure

The data were collected between 2009 and 2011 in the Hospital District of Helsinki and Uusimaa, which is located in Southern Finland, and comprises approximately 1.4 million inhabitants of whom ca. 82 500 were 13- to 17-year-old adolescents. This hospital district has three rehabilitation units for adolescents with schizophrenia. The study comprised a consecutive sample of patients from all these rehabilitation units (n =18, 7 males, 11 females, mean age 15.6 years, SD 1.4, range 13–17) and their families. The patients exhibited neither comorbid substance use or mood disorders nor somatic or neurological disorders. The clinical diagnoses of schizophrenia were confirmed with the Schedule for Affective Disorders and Schizophrenia for School-Aged Children – Present and Life-Time (K-SADS-PL) for the DSM-IV [22], performed by clinical adolescent psychiatrist trained for this assessment. Four persons suffered from childhood-onset schizophrenia, whereas among the others, onset of the disorder occurred at age 13 or later in life. Altogether 17 participants scored an IQ ≥ 70 (mean 93, SD 16) in a standard psychological assessment (WISC-III/WAIS-III) performed during or before the study period. Based on life chart information, all participants showed a primary IQ in normal range. One adolescent had suffered from epilepsy in early childhood, but was seizure-free and without antiepileptic medication during the study period. A non-contrast brain MRI was performed in all participants and evaluated by a clinical radiologist. The result was normal in all cases. Ten adolescents who were suffering from schizophrenia had no comorbid PDDs. Of the eight participants with PDDs, two suffered from childhood autism (F84.0), three from atypical autism (F84.1), and three from Asperger syndrome (F84.5). We have previously published a study using the same sample of patients. For details, see Waris et al. [23].

### Methods

#### Background information

Of the 18 participants, birth records were available for 16, records from well-baby clinics for 15, and school health care reports for 17.

#### DISCO

The Diagnostic Interview for Social and Communication Disorders (DISCO) version 11 developed by Wing and Gould [24] is a semi-structured interview that is administered to parents or other primary caregivers. The interview takes two to three hours and contains 387 questions about current skills, the development of these skills, and atypical behavior (e.g. repetitive stereotyped activities, emotions, maladaptive behavior and psychiatric disorders and forensic problems) during the lifespan of the adolescents. The DISCO is designed for both clinical and research use to assess autism spectrum disorders in individuals of all ages and ability levels; a computerized algorithm was developed for this latter purpose. The DISCO has good inter-rater reliability [25] and high agreement between the DISCO and the Autism Diagnostic Interview- Revised (ADI-R) classification has also been reported [26]. In the present study, one of the authors (P.W.), trained in the use of the DISCO assessment, interviewed all participants’ parents.

Twenty eight specific items from the DISCO interview were chosen to assess catatonic features in line with a previous study by Wing and Shah [11] (see Table 1). Each item was defined as either absent or present. In the original DISCO manual, the items which are present are divided into moderate or marked according to their severity. However, in line with the study by Wing and Shah, this was not done in the present study. Moreover, in the present study, the catatonic features were evaluated as lifetime features, features existing before the age of 10 (=childhood catatonic features) and features existing at the age of 10 or later in life (=adolescence catatonic features).

**Table 1:** Childhood (<10 Years of Age), adolescence (>10 Years of Age) and lifetime prevalence of catatonic features in 18 youngsters suffering from Schizophrenia

**Feature**	**Childhood**	**Adolescence**	**Lifetime**
**Movement**			
Odd gait	7/18 (38.9%)	5/18 (27.8%)	8/18 (44.4%)
Poor coordination	4/18 (22.2%)	7/18 (38.9%)	8/18 (44.4%)
Odd hand postures	0/18 (0.0%)	2/18 (11.1%)	2/18 (11.1%)
Runs in circles	0/18 (0.0%)	0/18 (0.0%)	0/18 (0.0%)
Rocks while sitting	2/18 (11.1%)	7/18 (38.9%)	8/18 (44.4%)
Complex body movements	0/18 (0.0%)	4/18 (22.2%)	4/18 (22.2%)
Walks on tiptoe	2/18 (11.1%)	1/18 (5.6%)	2/18 (11.1%)
Grimaces	2/18 (11.1%)	4/18 (22.2%)	5/18 (27.8%)
Lacks facial expression	4/18 (22.2%)	17/18 (94.4%)	17/18 (94.4%)
**Speech and vocalization**			
Immediate echolalia	2/18 (11.1%)	0/18 (0.0%)	2/18 (11.1%)
Delayed echolalia	4/18 (22.2%)	5/18 (27.8%)	8/18 (44.4%)
Odd intonation	4/18 (22.2%)	14/18 (77.8%)	14/18 (77.8%)
Shrieks for no reason	1/18 (5.6%)	3/18 (16.7%)	3/18 (16.7%)
Laughs for no reason	0/18 (0.0%)	8/18 (44.4%)	8/18 (44.4%)
**Behavior**			
*Eye contact*			
Poor eye contact	5/18 (27.8%)	14/18 (77.8%)	14/18 (77.8%)
Sideways glances	2/18 (11.1%)	5/18 (27.8%)	5/18 (27.8%)
Blank look in eyes	2/18 (11.1%)	9/18 (50.0%)	10/18 (55.6%)
Stares	3/18 (16.7%)	7/18 (38.9%)	7/18 (38.9%)
*Visual fascinations*			
Spins objects	0/18 (0.0%)	0/18 (0.0%)	0/18 (0.0%)
Twists hands near eyes	0/18 (0.0%)	0/18 (0.0%)	0/18 (0.0%)
Closely inspects objects from different angles	0/18 (0.0%)	0/18 (0.0%)	0/18 (0.0%)
*Unsocial behavior*			
Shouts for no reason	5/18 (27.8%)	8/18 (44.4%)	8/18 (44.4%)
Aggressive for no reason	2/18 (11.1%)	8/18 (44.4%)	8/18 (44.4%)
Lack of cooperation	8/18 (44.4%)	14/18 (77.8%)	15/18 (83.3%)
Destructive	6/18 (33.3%)	7/18 (38.9%)	10/18 (55.6%)
Strips in public	4/18 (22.2%)	6/18 (33.3%)	6/18 (33.3%)
Inappropriate personal habits	1/18 (5.6%)	1/18 (5.6%)	1/18 (5.6%)
Hyperactive	1/18 (5.6%)	3/18 (16.7%)	4/18 (22.2%)

### Ethics

After receiving verbal and written information about the study, all participants and their parents provided their written informed consent. The study plan was scrutinized by the Ethics Committee of the Helsinki and Uusimaa Hospital Districts. The permission to conduct the study was granted by the pertinent institutional authorities of the Helsinki University Central Hospital and Hyvinkää Hospital Area.

### Statistics

The Mann–Whitney *U*-test, Likelihood ratio chi square-test, and Fisher’s exact test were used to compare the groups. The Wilcoxon signed rank test for within group comparisons was used. The findings were considered significant when p- values < 0.05 were obtained. The magnitude of the effect size (phi coefficient) was interpreted as follows: 0.00 to 0.10, negligible association; 0.10 to 0.20, weak association; 0.20 to 0.40, moderate association; 0.40 to 0.60, relatively strong association; 0.60 to 0.80, strong association; and 0.80 to 1.00, very strong association [27]. The data were analyzed using SPSS version 21.0 (Macintosh) [28].

## Results

### The whole study group

All 18 adolescents with schizophrenia had showed some lifetime catatonic features. Fourteen of them (77.8%) had already expressed these features before the age of 10. The number of catatonic features increased significantly in transition from childhood to adolescence (in childhood: mean 4.1, SD 4.40, median 3, range 0–14, vs. in adolescence: mean 8.9, SD 3.07, median 9, range 4–15; Wilcoxon rank signed test, z = -3.249, p = 0.001).

The childhood, adolescence and lifetime prevalence of 28 catatonic features are presented in Table 1. The most prevalent childhood catatonic features were “lack of cooperation”, “odd gait”, “destructive behavior”, “shouting for no reason” and “poor eye contact”, in that order. In adolescence, the most prevalent features were “lack of facial expression”, “lack of cooperation”, “poor eye contact”, “odd intonation” and “blank look in eyes”. The most prevalent lifetime catatonic features were “lack of facial expression”, “lack of cooperation”, “odd intonation”, and “poor eye contact” followed by “blank look in eyes” and “destructive behavior”.

### Comparisons between the adolescents with a comorbid PDD and those without one

#### The number of catatonic features in childhood

The number of catatonic features was significantly higher among the patients with a comorbid PDD compared to those without one (patients with PDD: mean 7.3, SD 4.71, median 7, range 0–14, vs. patients without PDD: mean 1.5, SD 1.72, median 1, range 0–5, U = 12, p = 0.01).

Of the 28 catatonic features studied, a total of 22 different features (78.6%) occurred in patients with schizophrenia and a comorbid PDD. Among schizophrenia patients without a comorbid PDD, only 11 (39.3%) different features occurred. The difference between the groups was significant (*x*^2^ = 11.61, p = <0.001).

#### The number of catatonic features in adolescence

The number of catatonic features did not significantly differ between the two groups (patients with a comorbid PDD: mean 9.4, SD 3.34, median 9.5, range 5–15, vs. patients without a comorbid PDD: mean 8.5, SD 2.95, median 9, range 4–13, U = 34, p = NS).

Of the 28 catatonic features studied, a total of 22 different features (78.6%) occurred in patients with schizophrenia and a comorbid PDD. Among the patients without a comorbid PDD, only 19 (67.9%) different features occurred. The difference between the groups was significant (*x*^2^ = 4.18, p = 0.04).

#### The number of lifetime catatonic features

The number of lifetime catatonic features did not significantly differ between the two groups (patients with a comorbid PDD: mean 11.1, SD 3.09, median 11, range 6–16, vs. patients without a comorbid PDD: mean 8.8, SD 2.94, median 9, range 4–14, U = 22.50, p = NS).

Of the 28 catatonic features studied, a total of 24 different features (85.7%) occurred in patients with schizophrenia and a comorbid PDD. Among schizophrenia patients without a comorbid PDD, 21 (75.0%) different features occurred. The difference was not statistically significant [*x*^2^ = 2.31, p = NS).

#### Transition from childhood to adolescence

Among the patients with a comorbid PDD, the number of catatonic features did not significantly increase in transition from childhood to adolescence (in childhood: mean 7.3, SD 4.71, median 7, range 0–14 vs. in adolescence: mean 9.4, SD 3.34, median 9.5, range 5–15; Wilcoxon rank signed test, z = -1.532, p = NS). In contrast, the number of catatonic features increased significantly in transition from childhood to adolescence among those without a comorbid PDD (in childhood: mean 1.5, SD 1.72, median 1, range 0–5 vs. in adolescence: mean 8.5, SD 2.95, median 9, range 4–13; Wilcoxon rank signed test, z = -2.809, p = 0.005).

#### Prevalence of specific catatonic features

The comparisons of the frequencies of 28 catatonic features in childhood between the two groups are presented in Table 2. There were five specific features; “lacks facial expression”, “delayed echolalia”, “odd intonation”, “destructive” and “strips in public” that occurred significantly more often among the patients with a comorbid PDD.

**Table 2:** Prevalence of childhood (<10 Years of Age), adolescence (≥10 Years of Age), and lifetime catatonic features among adolescent Schizophrenia patients with (n = 8) and without (n =10) a History of ChildhoodPervasive Developmental Disorder (PDD)

	**Childhood**				**Adolescence**				**Lifetime**			
**Feature**	**PDD+**	**PDD-**	**p**	**Phi**	**PDD+**	**PDD-**	**p**	**Phi**	**PDD+**	**PDD-**	**p**	**Phi**
**Movement**												
Odd gait	62.5%	20.0%	NS		37.5%	20.0%	NS		62.5%	30.0%	NS	
Poor coordination	37.5%	10.0%	NS		37.5%	50.0%	NS		37.5%	50.0%	NS	
Odd hand postures	0.0%	0.0%	NS		12.5%	10.0%	NS		12.5%	10.0%	NS	
Runs in circles	0.0%	0.0%	NS		0.0%	0.0%	NS		0.0%	0.0%	NS	
Rocks while sitting	25.0%	0.0%	NS		37.5%	40.0%	NS		50.0%	40.0%	NS	
Complex body movements	0.0%	0.0%	NS		25.0%	20.0%	NS		25.0%	20.0%	NS	
Walks on tiptoe	12.5%	10.0%	NS		12.5%	0.0%	NS		12.5%	10.0%	NS	
Grimaces	12.5%	10.0%	NS		50.0%	0.0%	0.02	0.60	50.0%	10.0%	NS	
Lacks facial expression	50.0%	0.0%	0.02	0.60	87.5%	100.0%	NS		87.5%	100.0%	NS	
**Speech and vocalization**												
Immediate echolalia	25.0%	0.0%	NS		0.0%	0.0%	NS		25.0%	0.0%	NS	
Delayed echolalia	62.5%	0.0%	0.007	0.69	37.5%	20.0%	NS		75.0%	20.0%	NS	
Odd intonation	50.0%	0.0%	0.02	0.60	75.0%	80.0%	NS		75.0%	80.0%	NS	
Shrieks for no reason	12.5%	0.0%	NS		37.5%	0.0%	NS		37.5%	0.0%	NS	
Laughs for no reason	12.5%	0.0%	NS		50.0%	40.0%	NS		50.0%	40.0%	NS	
**Behavior**												
*Eye contact*												
Poor eye contact	50.0%	10.0%	NS		87.5%	70.0%	NS		87.5%	70.0%	NS	
Sideways glances	12.5%	10.0%	NS		25.0%	30.0%	NS		25.0%	30.0%	NS	
Blank look in eyes	25.0%	0.0%	NS		37.5%	60.0%	NS		50.0%	60.0%	NS	
Stares	25.0%	10.0%	NS		37.5%	40.0%	NS		37.5%	40.0%	NS	
*Visual fascinations*												
Spins objects	0.0%	0.0%	NS		0.0%	0.0%	NS		0.0%	0.0%	NS	
Twists hands near eyes	0.0%	0.0%	NS		0.0%	0.0%	NS		0.0%	0.0%	NS	
Closely inspects objects from different angles	0.0%	0.0%	NS		0.0%	0.0%	NS		0.0%	0.0%	NS	
*Unsocial behavior*												
Shouts for no reason	37.5%	20.0%	NS		50.0%	40.0%	NS		50.0%	40.0%	NS	
Aggressive for no reason	12.5%	10.0%	NS		37.5%	50.0%	NS		37.5%	50.0%	NS	
Lack of cooperation	62.5%	30.0%	NS		62.5%	90.0%	NS		75.0%	90.0%	NS	
Destructive	62.5%	10.0%	0.04	0.55	37.5%	40.0%	NS		75.0%	40.0%	NS	
Strips in public	50.0%	10.0%	0.02	0.60	50.0%	20.0%	NS		50.0%	25.0%	NS	
Inappropriate personal habits	12.5%	0.0%	NS		12.5%	0.0%	NS		12.5%	0.0%	NS	
Hyperactive	12.5%	0.0%	NS		0.0%	30.0%	NS		12.5%	30.0%	NS	

The Fishers’ Exact Test Was Used to Compare the Groups. Effect Sizes (Phi) Are Reported.

The comparisons of the frequencies of 28 catatonic features in adolescence between the two groups are presented in Table 2. There was only one catatonic feature, “grimaces”, that occurred significantly more often among the patients with a comorbid PDD.

The group comparisons for lifetime catatonic features are presented in Table 2. No statistically significant differences were observed.

## Discussion

Our first hypothesis states that adolescents suffering from schizophrenia show catatonic features. This hypothesis was verified since the entire study group had presented many lifetime catatonic features. This finding is in line with the previous studies by Thakur et al. [13] and Green et al. [14].

Our second hypothesis states that catatonic features are more prevalent among adolescent schizophrenia patients with a comorbid PDD than those without one. We found that this was true in childhood but was no longer the case in adolescence. Wing and Shah [11] chose their 28 specific items on the bases of clinical similarities between catatonia-like behavior and the unusual patterns of movement, speech, and behavior found in persons with autism spectrum disorders. These features are typically present already in early childhood and they have a tendency to become less marked with increasing age, especially in more able persons. In the present study, the number of catatonic features did not significantly change in transition from childhood to adolescence among the patients with a comorbid PDD. Among those without this comorbidity, the number of catatonic features increased significantly in transition from childhood to adolescence. In the future, it would be interesting to study whether this reflects the onset and impact of the schizophrenia process, although other factors, e.g. antipsychotic medication, may also contribute to this result.

More detailed analysis revealed that during their childhood, schizophrenia patients with a comorbid PDD differed from those without one in five specific items: “lacks facial expression”, “delayed echolalia”, “odd intonation”, “destructive behavior” and “strips in public”. This finding is, at least partially, a circular argument. “Lacks facial expression” and “stripping in public” can be regarded as qualitative impairments in reciprocal social interactions and “delayed echolalia” and “odd intonation” as qualitative impairments of verbal and non-verbal communication. Patients with PDDs also exhibit repetitive and stereotyped patterns of behaviour, and when those are prevented the person may get very anxious and sometimes even destructive. Thus, there is an overlap between catatonic features by Wing and Shah [11] and the core symptoms of PDDs.

Over three-quarters of our patients shared four catatonic features during their lifetime: “lacks facial expression”, “odd intonation”, “poor eye contact” and “lack of cooperation”. On the other hand, none of our patients exhibited any features from the “visual fascination” subgroup. Wing and Shah [11] reported that over three-quarters of their patients with autistic spectrum disorders shared these same four catatonic features plus the feature called “delayed echolalia”. The authors also reported that the frequencies of features in the “visual fascination” subgroup were very low. Given the fact that the authors had a sample of 200 autistic persons of whom a substantial proportion (35%) suffered also from mental retardation, it is interesting to note this overlap between their and our results.

In a study by Wing and Shah [29] about one in seven patients with autism spectrum disorders fulfilled the clinical diagnosis of catatonia. Kakooza-Mwezine et al. [30] have noted that the risk of developing clinical catatonia is about the same in persons with autism as in those suffering from affective and psychotic disorders. Further studies with follow-ups from childhood to adulthood are needed to find out whether differences in the occurrence of clinical catatonia as conceptualized in the DSM-5 among schizophrenia patients with and without a comorbid PDD will emerge. The same naturally applies to catatonia-like features and their waxing or waning or possible exacerbation into clinical catatonia.

The strengths of our study include recruiting patients on a consecutive basis from inpatient units in a geographically defined area. In addition, structured instruments with good psychometric properties were used in combination with primary documents from early childhood, which are readily available in Finland. Nevertheless, the study has its obvious limitations. The small sample size reduces the generalizability of our findings and the results must be regarded as preliminary. Moreover, the current study may have been underpowered to detect smaller statistical group differences that could have been detected in a larger sample. Further, our adaptation of the DISCO interview by splitting it into childhood and adolescence sections has not been validated. However, our use of the DISCO was in accordance with the original structure and design of this diagnostic instrument.

## Conclusions

Adolescent schizophrenia patients with a comorbid PDD show many catatonic features in childhood whereas those without one seem to develop these features first in adolescence. Catatonic features exhibited by adolescents with schizophrenia resemble those described among persons suffering from PDDs without schizophrenia.

## Competing interests

The authors declare no conflict of interest.

## Authors’ contributions

PW planned the study, collected, organized and analyzed the data and served as the first author. NL participated in the planning, analysis and writing processes. KK participated in collecting the data. JL performed statistical analyzes. PT supervised the study project and participated in the writing process. All authors read and approved the final manuscript.
